# The use of genetic markers to estimate relationships between dogs in the course of criminal investigations

**DOI:** 10.1186/s13104-017-2722-6

**Published:** 2017-08-17

**Authors:** Roberta Ciampolini, Francesca Cecchi, Isabella Spinetti, Anna Rocchi, Filippo Biscarini

**Affiliations:** 1 Dipartimento di Scienze Veterinarie, Università di Pisa, V.le delle Piagge 2, 56124 Pisa, Italy; 2 Dipartimento di Patologia Chirurgica, Medica, Molecolare e dell’Area Clinica, Università di Pisa, Via Savi 10, 56126 Pisa, Italy; 3 CNR-IBBA, Via Bassini 15, 20133 Milano, Italy; 4 Division of Infection & Immunity, School of Medicine, Cardiff University, Heath Park, Cardiff, CF14 4XN UK

**Keywords:** Forensic science, Genetic analysis, Dog, Fatal aggression, Molecular markers, Genotyping, Microsatellites, Relatedness

## Abstract

**Objective:**

Attacks on humans by dogs in a pack, though uncommon, do happen, and result in severe, sometimes fatal, injuries. We describe the role that canine genetic markers played during the investigation of a fatal dog-pack attack involving a 50-year-old male truck driver in a parking lot in Tuscany (Italy). Using canine specific STR genetic markers, the local authorities, in the course of their investigations, reconstructed the genetic relationships between the dogs that caused the deadly aggression and other dogs belonging to the owner of the parking who, at the moment of the aggression, was located in another region of Italy.

**Results:**

From a Bayesian clustering algorithm, the most likely number of clusters was two. The average relatedness among the dogs responsible for the aggression was higher than the average relatedness among the other dogs or between the two groups. Taken together, all these results indicate that the two groups of dogs are clearly distinct. Genetic relationships showed that the two groups of dogs were not related. It was therefore unlikely that the murderous dogs belonged to the owner of the parking lot who, on grounds of this and additional evidence, was eventually acquitted.

**Electronic supplementary material:**

The online version of this article (doi:10.1186/s13104-017-2722-6) contains supplementary material, which is available to authorized users.

## Introduction

Dog-pack attacks on humans are uncommon but may result in severe and sometimes fatal injuries. Although individually benign, dogs in a group can become aggressive, and attacks may occur. These may sometimes have judicial implications, and official investigations may ensue.

Science and technology have long been used to aid investigations (if a brief digression into crime fiction is allowed, Sherlock Holmes based his deduction process on the careful examination of clues thus providing inspiration to forensic science [1, 2]). Molecular biology is a powerful tool to help collect evidence and guide decisions in legal trials. The analysis of DNA is a consolidated technique in forensics: it is used for personal identification (e.g to confirm or exclude suspects based), to settle family controversies (e.g. parentage verification), to track the origin of pollen or other plant materials detected on the victim or alleged culprit [3, 4]. Genomics has therefore revolutionised the field of forensics, and this holds also for cases where animals are involved. In elephants, microsatellites have been used to identify the origin of ivory samples and tackle illegal trade [5]. In dogs, individual identification using short tandem repeats (STRs) is becoming common in solving criminal cases [6–9]. Microsatellite markers are useful for estimating the genetic relatedness between individuals of unknown ancestry, which is especially important when there are no genealogical data—unlike pure breeds for which pedigree is available and relatedness can be estimated from genealogies [10, 11].

This article presents the use of STR markers to estimate the genetic relatedness of dogs pertinent to the investigation of the death of a 50 year-old man on the yard of a transport company. The owner of the company had a number of dogs, and it was hypothesized that the dogs responsible of the fatal assault belonged to him. The authorities in charge of the case considered it important for the investigations to precisely identify the dogs and assess any family relationships between the killer dogs and the dogs registered under the name of the suspect.

## Main text

### Background of the case

In February 2012, in Tuscany (Italy), a 50-year-old male truck driver was found dead by his colleagues in the yard of a car transport company. On the ground of the yard, the police found traces of blood, pieces of clothes and shoes scattered everywhere, and the obvious signs of an attempt to escape of the truck driver. There was evidence (scraps of clothes of the victim found in the digestive tract) that eight dogs were responsible for the fatal attack. These dogs were handed over to veterinary services to be confined in a municipal dog pound. The dogs were half-breed Doberman/Rottweiler of medium/large size, and stationed in the parking lot used by the truck firm. From witnesses reports, a colleague of the victim confirmed that the presence of the dogs in the parking lot was known to all workers of the transport company—who saw them loitering around on an every-day basis— except the victim. The investigators found out that the owner of the parking lot owned a pack of dogs, half-breed Rottweiler/German-shepherd; most of these dogs were kept in his home-town (in Sicily), but exchange of dogs between the two places did occur occasionally. The investigators therefore suspected that the killer dogs might belong to the owner of the parking area, who would carry legal responsibility of leaving his dogs astray and, indirectly, of the deadly assault to the truck driver.

The investigators thought of reconstructing the relationships between animals, in order to understand if there were kinship relationships among the dogs that caused the fatal aggression, on one hand, and among these dogs and the dogs belonging to the owner of the parking lot, on the other hand, thus providing a possible link with the suspect. The Faculty of Veterinary Medicine of the University of Pisa was officialy appointed to provide an expert report on relationships among the dogs. All data were obtained as part of the criminal proceedings. Additional details can be found in the verdict and trial transcripts [12].

### Animals, DNA processing and genetic analysis

Blood samples were collected by officers of the public veterinary services from ten dogs and used for genetic investigation. Of these, eight samples came from the dogs found on the scene of the crime (group “Culprits”, 5 males—IDs 1, 4, 5, 7, 8—and 3 females—IDs 2, 3, 6), and the other two from phenotypically similar dogs, registered under the name of the owner of the parking lot where the crime took place, but kept elsewhere (group “Suspects”, both males—IDs 9, 10).

Genomic DNA was extracted (GenElute Blood Mammalian Genomic DNA Miniprep Kit^®^ kit, SGMA-ALDRICH Biotechnoloy USA) and subjected to spectrophotometric reading (NanoDrop Spectrophotometer—ND100^®^ Thermo Fisher Scientific Inc. USA) to assess quantity and quality (DNA between 25 and 70ng/μl, with a purity score between 1.5 and 1.8—absorbance ratio between 260 and 280 nm wavelength: a ratio of ~1.8 is generally considered to indicate good DNA purity [13, 14]).

The 19 microsatellites used in the analysis (Table 1) were located on 18 of the 39 chromosomes of the dog genome (*Canis lupus familiaris*); they are included in the “core panel” of loci recommended by the international society for animal genetics (ISAG) for parentage verification in the international canine comparison test [15]. The 19 microsatellites were co-amplified in a single multiplex PCR reaction. One primer from each pair was end-labeled with a fluorescent dye. After PCR, fragments were separated and detected via electrophoresis (ABI Prism 310 Genetic Analyzer, Applied Biosystems). DNA fragment size was analyzed with the GeneScanTM 500 LIZ size standard and GeneMapper analysis version 4.0 software (Applied Biosystems). Amplification and electrophoresis were repeated three times to verify the reproducibility of allele calling. The Amelogenin locus was included in the kit and used exclusively for sex determination (excluded from the genetic profile).

**Table 1:** STR markers used for sex determination (Amelogenin), the individual identification of the dogs, and the estimation of genetic relatedness among them

Marker	Chr	Repeat motif	Size range (bp)
FH2848	2	di	222–244
AHTh171	6	di	215–239
REN162C04	7	di	192–212
INU055	10	di	190–216
AHT137	11	di	126–156
FH2054	12	tetra	135–179
INU030	12	di	139–157
AHT121	13	di	68–118
REN169D01	14	di	199–221
REN247M23	15	di	258–282
AHTh260	16	di	230–254
REN54P11	18	di	222–244
INRA21	21	di	87–111
CXX279	22	di	109–13
AHTk253	23	di	277–297
AHTk211	26	di	79–101
REN169O18	29	di	150–170
INU005	33	di	102–136
Amelogenin	X		174–218

*Chr* Chromosome of the dog genome; Repeat motif: two (“di”) or four (“tetra”) base-pairs motifs

A cluster analysis using the Bayesian clustering algorithm implemented in the *STRUCTURE* v2.2 [16] was performed. *STRUCTURE* attempts to infer the most likely number of clusters (*K*) in which the population can be subdivided. Values of $K \in [1, 10]$ were tested ($10^6$ iterations—$10^4$ for burn-in). The analysis was repeated three times for each *K*. Optimal *K* was chosen from:

1 $$ \Delta K = \frac{\overline{ln\left(L''(K)\right)}}{\sqrt{Var(ln\left(L\left(K)\right)\right)}} $$

where *L*(*K*) is the likelihood of the *STRUCTURE* model $\forall K \in [1,10]$; $L''(K)$ is the second order rate of change of *L*(*K*) ($L'(K) = L(K) - L(K-1)$; $L''(K) = L'(K) - L'(K-1)$); *ln*() is the natural logarithm; average and variance refer to the three repetitions for each tested *K*. The *K* value corresponding to $max(\Delta K)$ gives the estimated number of clusters in the population [17]. Individual probabilities of belonging to each of the *K* clusters (*P*(*K*)) were obtained from *STRUCTURE*; the threshold value for assigning individuals to a given cluster was set at 0.90 [18].

Based on genotypes at the 18 STR loci listed in Table 1—except Amelogenin, maximum likelihood estimates of the genetic relatedness *r* [19] among dogs within and across groups (“Culprits” and “Suspects”) were obtained (*ML-RELATE* [20] and *KINGROUP* v.2 [21]). For each dog, the average pairwise relatedness with dogs from either groups was calculated.

Furthermore, genetic similarities between animals were investigated by comparing individual multilocus genotypes with each other [22]. Genetic similarity was defined as $P={}^{A}\!/_{2L}$, where *P* is the proportion of common alleles (*A*) in relation to the 2*L* possible alleles (*L* = number of loci). The similarities between each pair of individuals were then averaged over the whole population.

The comparison of multilocus genotypes is commonly performed in the context of the ISAG-international canine comparison test [23, 24]. The multilocus genotypes of the ten dogs of this study were compared to those of a random sample of 10 pure-breed dogs (assorted breeds) from the ISAG-international canine comparison test panel 2012 (CT2012). The results from a panel of 23 dogs from different pure breeds analyzed in the context of the ISAG-international canine comparison test panel 2008 and 2010 were also considered (CT2008, CT2010). This was done to provide a benchmark for lack of relatedness, against which relationships estimated between the dogs included in the study were compared.

### Results

**Fig. 1:**
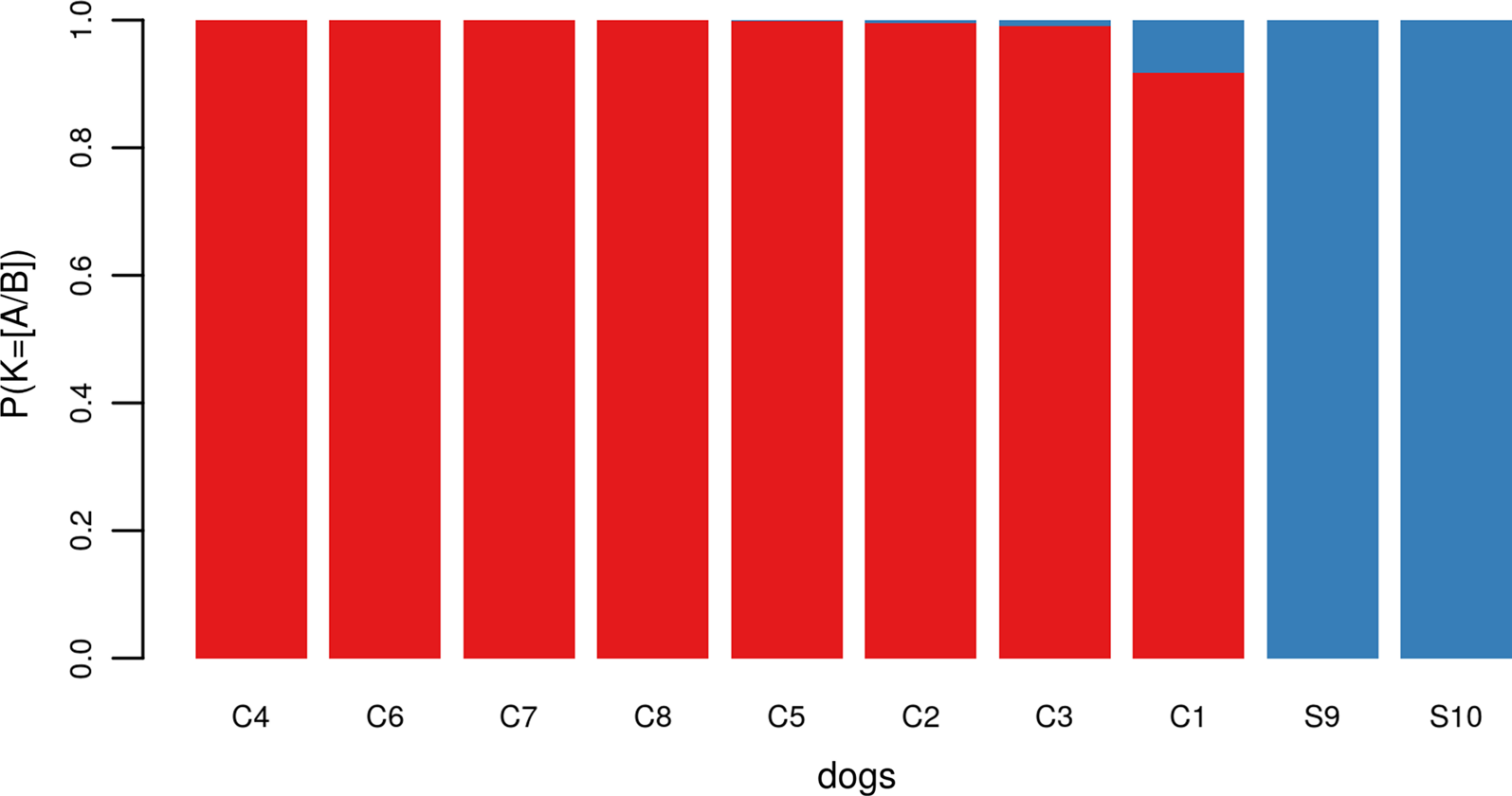
Probability of assignment of the dogs to clusters A (*green*) or B (*red*) ($P(K)=[A/B]$). Probability were obtained based on STR genotypes from the Bayesian clustering algorithm implemented in the software *STRUCTURE*

The Bayesian clustering algorithm gave the highest posterior probability for $K=2$ clusters. For each of the ten dogs, the probabilities of assignment to the two clusters were calculated. All dogs belonging to the “Culprits” group were assigned to Cluster *A*, while the two dogs belonging to the “Suspects” group were assigned to Cluster *B* (Fig. 1). Probabilities of assignment were always >0.99, except for dog C1, whose probability of belonging to cluster A was 0.92.

These results indicate that the two groups of dogs—those who attacked the victim and those belonging to the suspect—form two genetically distinct clusters. Furthermore, within the “Culprits” group dogs showed high values of pairwise relatedness (Additional file 1: between $-0.078$ and 0.147), while much lower relatedness was estimated with the two dogs from the “Suspects” group (lower than $-0.298$). It seems therefore unlikely that the dogs of the two groups are related.

**Fig. 2:**
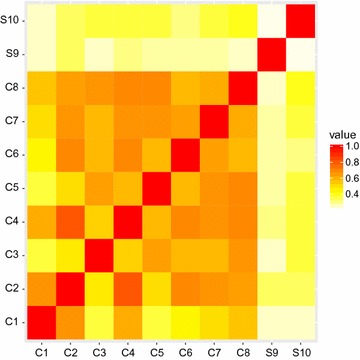
Heatmap of the proportion of alleles shared by dogs from both the culprits and the suspects groups (*darker colors* indicate closer genetic relatedness). Relatedness between dogs were estimated by comparing individual multilocus genotypes

From the multilocus genotype analysis, dogs of the “Culprits” group showed average similarity equal to 0.592 ($\pm 0.101$), ranging from 0.361 to 0.805 (Additional file 2). All Culprits dog shared at least 1 or 2 alleles at each locus. The sampled data from CT2012 showed that dogs of different breeds have an average genetic similarity of 0.246 ($\pm 0.061$), with minimum of 0.083 and maximum of 0.361. Similar values were observed in the CT2008 and CT2010 results (average similarity 0.285 [25]; see Additional file 3). The dogs used for the ISAG Comparisons are not relatives, but still show a moderate level of genetic similarity. This indicates that also individuals from different breeds may share a proportion of alleles, probably because of a common genetic basis across canine breeds. This is further supported by studies on representative samples of purebred dogs such as Bracco Italiano (average similarity 0.455) [26] and Pit-Bull Terrier (average similarity 0.412) [27]. The genetic similarity between “Culprits” and CT2012 dogs was 0.224 ($\pm 0.060$), ranging from 0.111 to 0.389. The average genetic similarities between the two groups (“Culprits” and “Suspects”) was low (0.169 ± 0.069). The heatmap of pairwise similarities (Fig. 2) illustrates how the “Culprits” dogs were related among themselves; no genetic relationships with the two“Suspects” dogs (unrelated between themselves) were apparent. All together, these results say that dogs from the “Culprits” group presented an average genetic similarity higher than what was found in unrelated populations, unlike dogs from the “Suspects” group, and that relations between the two groups were low.

The results from: (i) the analysis of population structure; (ii) the estimated genetic relatedness within- and across-groups; (iii) the multilocus genotype comparisons; and (iv) the proportion of STR alleles shared between dogs, have all been used to prepare the technical report needed by the judicial authorities during the trial (in Italian [12]). The strong family ties within the dogs implicated in the deadly assault, coupled with the lack of relationships between these and the dogs belonging to the suspect, were some of the elements that led to the acquittal of the owner of the parking lot where the accident took place.

These results fit in the broader context of the use of canine microsatellites in forensics, which has been repeatedly shown to be common, accurate and reliable [7, 9, 28, 29]. Applications of STR markers in dogs are not limited to forensics, but extend to, for example, parentage verification [30] and pedigree reconstruction [19, 31]. There is therefore intensive methodological research on the use of STR markers in dog genetics, aimed at improving techniques and at promoting standardization and harmonization across laboratories [32].

## Limitations

Molecular biology and the study of DNA can be very helpful in unravelling the unsolved issues of criminal cases. In the presented case, a man suspected of being indirectly responsible for the death of a truck driver caused by a pack of dogs, was acquitted also based on the evidence from genetic analysis. The dogs responsible of the attack were all half-breed Doberman/Rottweiler dogs, and the dogs belonging to the suspect were also half-breed, Rottweilwer/German-Shepherd. The dogs from the two groups were known to have potentially come into contact (the suspect did sometimes bring his dogs along when visiting the parking lot where the accident took place). The prosecutor therefore thought it reasonable to assess genetic relationships between dogs as one of the elements of the investigations. The incriminated dogs were shown not to be genetically related to similar dogs owned by the suspect and probably did not come from the same pack. However, it is also possible for unrelated dogs to live together and, viceversa, related dogs may live in different places. Further proof to show provenance from a common environment would be needed, like for instance the hair or skin microbiota profiling [33, 34].

We emphasize that results from genetic analysis were but one piece of the evidence [12] that contributed to relieve the suspect from the charge of having left his dogs astray and thus indirectly causing the death of the victim. We endeavoured to describe the genetic analysis as closely as possible to what was actually done in support of the official investigations. This constitutes an interesting example of the helpful use of genomics in forensics.

## Additional files

**Additional file 1.** Average pairwise molecular relatedness. Table of pairwise molecular relatedness between each dog and the dogs in either groups (“Culprits”, “Suspects”), estimated from STR markers.

**Additional file 2.** Genetic similarities. Table of genetic similarity among dogs from the multilocus genotype analysis.

**Additional file 3.** Average genetic similarities: this study and ISAG Canine Comparison Tests. Table of within-group average genetic similarity from the multilocus genotype analysis.

## Abbreviations

STRs: short tandem repeats
K: clusters
