# Supplementary material for: The use of genetic markers to estimate relationships between dogs in the course of criminal investigations
Source: BMC Res Notes. 2017 Aug 17;10:414. doi: 10.1186/s13104-017-2722-6 (PMC5561628; doi:10.1186/s13104-017-2722-6)
Supplement: Supplementary file 1 — Additional file 1. Average pairwise molecular relatedness. Table of pairwise molecular relatedness between each dog and the dogs in either groups (“Culprits”, “Suspects”), estimated from STR markers. [file 13104_2017_2722_MOESM1_ESM.pdf]

RESEARCH

The use of genetic markers to estimate relationships between dogs in the course of criminal investigations

Roberta Ciampolini, Francesca Cecchi, Isabella Spinetti, Anna Rocchi and Filippo Biscarini

Full list of author information is available at the end of the article

Table 1 Average pairwise molecular relatedness between each dog and the dogs in either groups ("Culprits", "Suspects"), estimated from STR markers

	Average pairwise relatedness	
Dog	vs Group Culprits	vs Group Suspects
Group Culprits		
C1	-0.078	-0.340
C2	0.064	-0.391
C3	0.025	-0.332
C4	0.147	-0.425
C5	0.101	-0.349
C6	0.119	-0.389
C7	0.142	-0.298
C8	0.140	-0.411
Group Suspects		
S9	-0.364	-0.228
S10	-0.369	-0.228

C1-8: the eight dogs belonging to the "Culprits" group; S9-10: the two dogs belonging to the "Suspects" group
