# Supplementary material for: The use of genetic markers to estimate relationships between dogs in the course of criminal investigations
Source: BMC Res Notes. 2017 Aug 17;10:414. doi: 10.1186/s13104-017-2722-6 (PMC5561628; doi:10.1186/s13104-017-2722-6)
Supplement: Supplementary file 2 — Additional file 2. Genetic similarities. Table of genetic similarity among dogs from the multilocus genotype analysis. [file 13104_2017_2722_MOESM2_ESM.pdf]

RESEARCH

The use of genetic markers to estimate relationships between dogs in the course of criminal investigations

Roberta Ciampolini, Francesca Cecchi, Isabella Spinetti, Anna Rocchi, Filippo Biscarini

Full list of author information is available at the end of the article

Table 1 Genetic similarity among dogs from the multilocus genotype analysis

Dog	C1	C2	C3	C4	C5	C6	C7	C8	S9
Group Culprits									
C1									
C2	0.667								
C3	0.361	0.472							
C4	0.611	0.805	0.528						
C5	0.361	0.500	0.639	0.583					
C6	0.444	0.694	0.583	0.694	0.583				
C7	0.500	0.661	0.583	0.666	0.667	0.638			
C8	0.555	0.639	0.661	0.694	0.694	0.583	0.611		
Group Suspects									
S9	0.250	0.333	0.250	0.305	0.278	0.278	0.278	0.250	
S10	0.250	0.333	0.361	0.361	0.361	0.305	0.361	0.389	0.222

C1-8: the eight dogs belonging to the "Culprits" group; S9-10: the two dogs belonging to the "Suspects" group
