# Supplementary material for: The use of genetic markers to estimate relationships between dogs in the course of criminal investigations
Source: BMC Res Notes. 2017 Aug 17;10:414. doi: 10.1186/s13104-017-2722-6 (PMC5561628; doi:10.1186/s13104-017-2722-6)
Supplement: Supplementary file 3 — Additional file 3. Average genetic similarities: this study and ISAG Canine Comparison Tests. Table of within-group average genetic similarity from the multilocus genotype analysis. [file 13104_2017_2722_MOESM3_ESM.pdf]

RESEARCH

The use of genetic markers to estimate relationships between dogs in the course of criminal investigations

Roberta Ciampolini, Francesca Cecchi, Isabella Spinetti, Anna Rocchi, Filippo Biscarini

Full list of author information is available at the end of the article

Table 1 Within-group average genetic similarity from the multilocus genotype analysis

Group	Avg Similarity
Culprits	0.592
Suspects	0.222
CT2008-CT2010	0.285
CT2012	0.246

Culprits: dogs responsible for the fatal attack; Suspects: dogs belonging to the suspected person; CT2008, CT2010, CT2012: random pure-bred dogs from the ISAG-International Canine Comparison Tests 2008-2010 (23 dogs) and 2012 (10 dogs).
